# Editorial: Equity in cancer care

**DOI:** 10.3389/fonc.2023.1346785

**Published:** 2024-01-05

**Authors:** Jorge J. Nieva

**Affiliations:** Department of Medicine, University of Southern California/Norris Comprehensive Cancer Center, Los Angeles, United States

**Keywords:** health equity, cancer care, cancer biology, global health, Social determinants of health

Different patient populations will experience different average outcomes from their cancer. This experience is not unique to any one nation or healthcare system but is seen worldwide and can result from population differences in economic factors, disease comorbidities, educational differences, and exposure to economic and political crises. Each of the manuscripts in this Research Topic represents an opportunity for improvement. After all, if we can see one group doing worse than another regarding cancer outcomes, it is relatively easy to imagine a solution that simply gives what the advantaged group has to those who lack it. In this Research Topic of Frontiers in Oncology dedicated to Equity in Cancer Care, we are presented with experiences worldwide that address these difficult problems. While seeing a solution may be easy, finding the resources to correct them is often where the challenge lies.

Sometimes the solution is simply to improve health literacy among women to improve rates of screening mammography (Poon et al.). But such interventions may be difficult to implement across populations where local effects of neighborhoods create heterogeneity in the population that call for unique approaches that may need to vary from block to block (Layne et al.). A better approach may be to alter the criteria for screening in the first place, to ensure that the indications for screening do not leave out minority groups (Olazagasti et al.). Meanwhile, segregation of populations can impact other health behaviors besides screening and ending such segregation may be a potential solution (Pichardo et al.), as targeted interventions aimed at minority populations have been notoriously difficult to conduct (Pichardo et al.). Simple solutions, such as making screening easier on patients, by covering sedation along with screening colonoscopy for example, may go a long way to improving screening rates among the poor (Zhuo et al.).

Other solutions may need to be rooted in biological differences between groups. A comorbidity such as diabetes and all its impacts on cancer care is not evenly distributed in patient populations and might make care for some groups inferior (Ashing et al.), though another study in this Research Topic found that for small cell lung cancer at least, those effects did not lead to inferior outcomes in minority populations at higher risk for diabetes (Olateju et al.). Cardiac disease is also unevenly distributed as a comorbidity in cancer patients and optimal outcomes for cancer will not be achieved if this disparity is not addressed (Patel et al.).

Biology matters. The cancer itself may also have different biology and there is much we can learn from studying differences among ethnic groups. Of course, if our cell lines come from only one segment of the population, we are missing a tremendous opportunity to have laboratory models that apply to the whole population of affected patients (Leon et al.). Cancer mediators such as miRNAs are differentially expressed among different ethnic groups and their study can offer insights into biology of neoplasia (Gobin et al.). The same can be said of genomic, epigenomic and transcriptomic signatures (Stevens et al.).

Sadly, regional, and national economic and political factors make the outcome of cancer worse. We have seen worsening of breast cancer mortality in South Africa despite the end of apartheid even as much of the rest of the world sees improvement (Olorunfemi et al.). In Lebanon, economic and policy factors have created an environment of drug shortages that have been devastating for cancer patients (Kattan and Kattan). Greater wisdom among leaders is badly needed and sometimes is in short supply. I hope that the insights from the excellent authors in this journal bring much wisdom to the readers and working together, we can make cancer outcomes better for everyone, starting with those who need it most.

## Author contributions

JN: Conceptualization, Project administration, Writing – original draft.

